# Advantages of the classical thermodynamic analysis of single—and multi-component Langmuir monolayers from molecules of biomedical importance—theory and applications

**DOI:** 10.1098/rsif.2023.0559

**Published:** 2024-01-10

**Authors:** Patrycja Dynarowicz-Latka, Anita Wnętrzak, Anna Chachaj-Brekiesz

**Affiliations:** Faculty of Chemistry, Jagiellonian University, Gronostajowa 2, 30-387 Kraków, Poland

**Keywords:** excess thermodynamic functions, Langmuir monolayers, air/water interface, interactions, phase transitions

## Abstract

The Langmuir monolayer technique has been successfully used for decades to model biological membranes and processes occurring at their interfaces. Classically, this method involves surface pressure measurements to study interactions within membrane components as well as between external bioactive molecules (e.g. drugs) and the membrane. In recent years, surface-sensitive techniques were developed to investigate monolayers *in situ*; however, the obtained results are in many cases insufficient for a full characterization of biomolecule-membrane interactions. As result, description of systems using parameters such as mixing or excess thermodynamic functions is still relevant, valuable and irreplaceable in biophysical research. This review article summarizes the theory of thermodynamics of single- and multi-component Langmuir monolayers. In addition, recent applications of this approach to characterize surface behaviour and interactions (e.g. orientation of bipolar molecules, drug-membrane affinity, lateral membrane heterogeneity) are presented.

## 1. Introduction

Most processes in nature take place at the boundary of two phases with different permittivity (*ε*). Among a variety of interfacial boundaries, the interface between air (*ε* = 1) and water (*ε* = 80) is the most common. Interestingly, its properties are similar to those found in living organisms, occurring at the junction of the lipid cell membrane (*ε* = 2) and the aqueous phase surrounding the cell (*ε* = 80). Therefore, due to the complexity and variability of natural systems, artificial membrane models (such as Langmuir monolayers formed at air/water interphase) provide a simplified but very useful platform that enables the systematic study of membrane interactions under controlled conditions [1,2]. Although monolayers represent half the natural bilayer membrane, under certain conditions both systems have similar behaviour. Already in 1979, Blume [3] showed, by comparing the change in surface area during the phase transition of a monolayer and a bilayer for a series of phospholipids, that both systems behave similarly at a surface pressure (π) of 30 mN m^−1^. This was confirmed in subsequent studies [4] and was called monolayer-bilayer correspondence [5–7], which was further evidenced in a number of experiments. Namely, studies on the enzymatic activity of phospholipase A2 in the monolayer compressed to 30 mN m^−1^ showed that it is identical to that in the bilayer [8,9]. Subsequently, it was shown that the lateral forces in Langmuir films at surface pressures from 30 to 35 mN m^−1^ are in the same range as in bilayers [4,10,11]. Later, based on LAURDAN generalized polarization function [12] it was reported that the similar molecular areas were occupied in bilayers and monolayers at surface pressure of 26 ± 2 mN m^−1^ (for DOPC) and 28 ± 3 mN m^−1^ (for DPPC). Additionally, based on X-ray measurements it was found that the molecular tilt of DPPC lipid chains (32°) and area (48 Å^2^/molecule) in monolayers at 35 mN m^−1^ [13,14] give very similar values as in fully hydrated bilayers (32^°^ and 47.2 Å^2^, respectively) [15]. This indicates that the hydration of monolayers and bilayers under these conditions is almost identical. However, it must be emphasized that the equivalence between monolayers and bilayers have been and still is a matter of debate [9].

**Figure 1.**
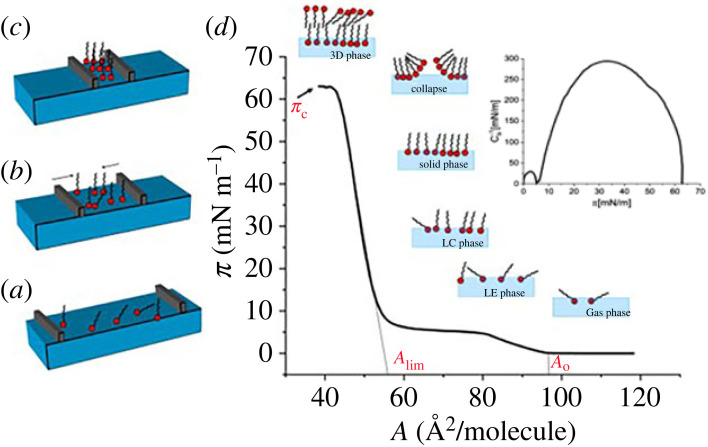
Schematic representation of Langmuir monolayer formation: (*a*) spreading; (*b*), (*c*) compression on a Langmuir trough; (*d*) the resultant surface pressure-area (*π–A*) isotherm on the example of DPPC. Inset- compression modulus ($C_{s}^{\text{–}1}$) versus surface pressure dependence.

The use of Langmuir films as biomembrane models results from the fact that the components of the cell membrane are surface-active due to their amphiphilic structure and are capable of forming monolayers at the free water surface. The advantages of using Langmuir monolayers result from easy and accurate control of the composition and packing of the mimicked membrane. This method is suitable for investigating the strength and nature of interactions (expressed as quantitative parameters of the interactions in terms of excess thermodynamic functions; see §2.2) but is not adequate for studying dynamics and transport across the membrane, for which other types of membrane models (e.g. liposomes) should be used. The simplest model of an artificial membrane modelled with the Langmuir technique would be a one-component monolayer, formed by one of the major membrane lipids, for example DPPC, or other selected lipid of interest; although most frequently a mixture of lipids characteristic of the investigated cellular membrane is used to construct an artificial membrane. The procedure involves spreading a chosen membrane lipid or lipid mixture dissolved in organic, volatile solvent immiscible with water (e.g. chloroform) at the air/water interface on a Langmuir trough (figure 1). After solvent evaporation, the surface is covered with monomolecular lipid film, the packing density of which can be easily changed by compressing the monolayer with sliding barrier(s), and the surface pressure is measured (usually with a Wilhelmy plate method) as a function of surface area per lipid molecule, yielding surface pressure–area per molecule (*π–A*) isotherms [16]. The obtained isotherm is characterized by the following parameters: *A*_0_, the lift-off area, which is the molecular area at the surface pressure rise; *A*_lim_, the limiting area, being the area extrapolated from the steepest part of the isotherm to π = 0; π_c_, the collapse pressure, which is the surface pressure at which monolayer collapses (discussed later on). From the isotherm datapoints, the compression modulus ($C_{s}^{\text{–}1}$) can be calculated (see §2.1). For a detailed description of the Langmuir films methodology, please see [1,17].

The development of techniques such as fluorescence microscopy [18], Brewster angle microscopy (BAM) [19], X-ray diffraction [20] and scattering [21], neutron reflectivity and scattering [22], vibrational spectroscopies (polarization-modulation infrared reflection-absorption spectroscopy, PM-IRRAS; sum frequency generation spectroscopy, SFG) [23,24] enabled the *in situ* characterization of monolayers, and helped to understand selected aspects of physiological action of biologically relevant molecules and nanomaterials [25]. Moreover, once monolayers are transferred to solid supports, a wider range of experimental methods become available [26–28]. However, the use of the above-mentioned techniques is not always conclusive, especially due to the poor signal-to-noise ratio or lack of reproducibility. By contrast, classical thermodynamic analysis always provides reliable results. Although a number of reviews on Langmuir monolayers have already been published [10,15,29–31], their scope is rather wide and general and does not meet the expectations of researchers seeking for verified protocols for biomolecule–membrane interactions study. Therefore, the aim of this work is to show in a concise way the advantages of the classical thermodynamic analysis, which has been for many years still very useful and irreplaceable in comprehensive biophysical analysis related to the characterization of the properties of bioactive substances.

## 2. Thermodynamic aspects of two-dimensional insoluble films

The surface behaviour of floating monolayers is convenient to describe in terms of differentiated integral state functions. Based on the first law of thermodynamics (written as dU=δq+δw) [32], the differential of the internal energy (*U*) and the other thermodynamic functions (*F*, Helmholtz free energy; *H*, enthalpy; *G*, Gibbs free energy, also called free enthalpy) of two-dimensional system may be expressed as [32]

2.1 $$dU(S,A)=TdS+\gamma dA={\left(\frac{\partial U}{\partial S}\right)}_{A}dS+{\left(\frac{\partial U}{\partial A}\right)}_{S}dA,$$

2.2 $$dF(T,A)=-\;SdT+\gamma dA={\left(\frac{\partial F}{\partial T}\right)}_{A}dT+{\left(\frac{\partial F}{\partial A}\right)}_{T}dA,$$

2.3 $$dH(S,\pi )=TdS-Ad\gamma ={\left(\frac{\partial H}{\partial S}\right)}_{\pi }dS+{\left(\frac{\partial H}{\partial \pi }\right)}_{S}d\pi$$

2.4 $$\begin{array}{r} dG(T,\pi )=-SdT-Ad\gamma ={\left(\frac{\partial G}{\partial T}\right)}_{\pi }dT+{\left(\frac{\partial G}{\partial \pi }\right)}_{T}d\pi . \end{array}$$

In the above equations, the surface pressure π (two-dimensional equivalent of osmotic pressure (*Π*) in three-dimensional systems) [32] is defined as the difference in interfacial tension (force per unit length acting on an imaginary line drawn on a surface) between the clean interface and the interface in the presence of a monolayer (*γ*_0_ − *γ*); therefore dπ = −d*γ*; *S* refers to entropy; *A* is an area (equivalent of volume (*V*) in two-dimensional systems), and *T* is temperature. Additionally, it is assumed that the amount of substance (expressed as number of moles, *n*) does not change (d*n* = 0). Using the above relationships, the surface tension can be redefined. In isothermal-isochoric conditions, the surface tension *γ* can be expressed as ${\left(\partial F/\partial A\right)}_{T,V,n}$; however, if the process takes place in isothermal-isobaric conditions, the surface tension is related to the change in the Gibbs free energy,

2.5 $$\gamma ={\left(\frac{\partial G}{\partial A}\right)}_{T,p,n}.$$

### 2.1. Single-component films

The result of the Langmuir monolayer experiment is the π–*A* isotherm (figure 1), which can be used to calculate the work associated with compressing the monomolecular film from the surface pressure π_1_ to π_2_. The value of this work includes the energy of compression of the monolayer with moving barriers along with the energy needed to transition the film from the expanded to condensed state [33]. Based on equation (2.4), the work of compression, *W*^comp^, can be attributed to the change in the Gibbs free energy during film compression under isothermal conditions,

2.6 $$W^{\mathrm{comp}}=\;\Delta G^{\mathrm{comp}}=N_{A}\int_{\pi_{1}}^{\pi_{2}}Ad\pi ,$$

where *N_A_* is the Avogadro number and *A* is area per molecule in one-component film. The work of expansion (*W*^exp^) can be calculated in a similar way. In many cases, the course of isotherms obtained as a result of expansion does not coincide with the results of compression, which suggests the irreversibility of the transition and the accumulation of energy in the resulting phase. The degree of hysteresis (*W*^hys^) can be quantified by the difference between *W*^exp^ and *W*^comp^; however, in this case, *W*^hys^ cannot be identified with ΔG (which is a state function, and its value cannot depend on the transition path). Additionally, the entropy change for hysteresis can be obtained from the following equations:

2.7 $${\left[\Delta S_{\pi }^{\mathrm{hys}}=R\ln\frac{A_{\exp}}{A_{\mathrm{comp}}}\right]}_{\pi }$$

and

2.8 $$\Delta S^{\mathrm{hys}}=\underset{\pi }{\sum }\Delta S_{\pi }^{\mathrm{hys}}.$$

Based on the π–*A* isotherm data, the parameters characterizing the film in-plane elasticity can be calculated and expressed as: compressibility (*C*_s_) or compressibility modulus, $C_{s}^{\text{–}1}$ (which is the reciprocal of the compressibility),

2.9 $$C_{s}^{-1}=-A{\left(\frac{\partial \pi }{\partial A}\right)}_{T}\;.$$

The $C_{s}^{\text{–}1}$ value reflects the packing state of the monolayer and indicates the physical state of the film at a particular stage of compression (gas (G), liquid expanded (LE), liquid condensed (LC), solid (S)) [10].

Molecules arranged in two-dimensional system can—just like bulk molecules—undergo organizational or conformational changes known as a phase transition. Such transitions of various nature are manifested as discontinuity in the π–*A* curves, appearing as (i) a change in slope (kink in the isotherm), or (ii) the appearance of a plateau region [34–36]. For many biomolecules phase transition occurs as a region of constant surface pressure (π_t_) observed upon the decrease in the area per molecule upon film compression (see the isotherm for DPPC, figure 1). It is assumed that at the beginning of the plateau, the initial phase starts to undergo the gradual transition to the second phase (which is fully developed at the end of the plateau). The process is isobaric and isothermal. Additionally, it can be assumed that the Gibbs free energy values of the two phases are equal as they are in equilibrium during a plateau. After rewriting equation (2.4) for phases existing at the beginning and at the end of the plateau and performing certain transformations, the following equation is obtained [32]:

2.10 $$\Delta S_{t}=\left(\left(\frac{\partial \pi_{t}}{\partial T}\right)-\left(\frac{\partial \gamma_{0}}{\partial T}\right)\right)\Delta A,$$

where $\left(\partial \pi_{t}/\partial T\right)$ is the slope of the dependence of the *π_t_*(*T*) (values obtained from *π–A* isotherms measured at different temperatures), and Δ*A* = *A*_e_ − *A*_b_, where *A*_e_ is the area per molecule at the end of plateau and A_b_ is the area per molecule at its beginning. The value of $\left(\partial \gamma_{0}/\partial T\right)$, where *γ*_0_ is the surface tension of pure water, is equal to −0.153 mN m^−1^ K^−1^ for temperatures between 10 and 35°C. Based on the obtained transition entropy values, the transition enthalpy can be calculated as

2.11 $$\Delta H_{t}=T\Delta S_{t}.$$

When a monolayer is compressed to the limit of its stability in two dimensions it assembles into three-dimensional structures, which can be connected with the *monolayer collapse*. This process is manifested as a spike or plateau appearing at the highest pressure (π_c_) in the course of the *π–A* isotherm. The value of π_c_ is an indicator of the film stability, namely the higher the value, the more stable the monolayer is. For details regarding the molecular mechanism and kinetics of collapse, we refer the readers to the review articles [37–39]. The mechanism of collapse generally depends on physical state of monolayers (liquid versus condensed). Indeed, fluid lipid monolayers collapse upon desorption of the film material into the subphase. In contrast, monolayers that are rigid and more ordered collapse by fracturing and subsequent loss of the material to the subphase, or formation of multi-layer structures. Moreover, the collapse of lipid monolayer may lead to the formation of different three-dimensional aggregates in the subphase, such as vesicles, micelles, tubes, twisted ribbons, discs or bilayer folds [40]. This fact is especially important in biomedical sciences. Also, the reversibility of collapse is of a special importance in many *in vivo* processes, for example in the lung, eyes and ears (discussed in more details in §3.2).

### 2.2. Multi-component films

The use of Langmuir monolayers in biological and medical sciences involves the study of complex systems that contain several components. Multi-component films can be analysed based on their mutual miscibility, which can be described analogously to miscibility in three-dimensional systems (i.e. the components may mix completely, partially, or not at all) [41]. In this treatment the presence of solvent molecules is ignored and the surface phase is considered—in the simplest case—as a system containing two film-forming components.

For miscible films, interactions between film-forming molecules can be estimated in two ways: (i) qualitative with additivity rules, and (ii) quantitative with excess functions (defined as the difference between values of the thermodynamic function for a real and ideal system).

The additivity rule is represented by the following equations [41,42]:

2.12 $$A_{1\ldots N}^{\mathrm{id}}=\sum_{i=1}^{N}A_{i}X_{i}$$

and

2.13 $$\pi_{c\;1\ldots N}^{\mathrm{id}}=\sum_{i=1}^{N}\pi_{c\;i}X_{i}.$$

These equations describe the area per molecule at a given surface pressure (or the surface pressure of monolayer collapse, respectively) as a function of film composition. The linear course of the above functions indicates the ideal miscibility of the components or their complete immiscibility, whereas deviations from linear dependence indicate non-ideality and interactions in these systems. In addition, in the case of immiscibility, two collapse states, corresponding to pure components, are visible in the course of the isotherm and their values do not change with film composition. On the contrary, for miscible films, the collapse occurs at the pressure in-between π_c_ for pure components and its value varies upon changing the proportion of film components. This is interpreted according to the phase rule (see [41] for details).

A more detailed description of mutual interactions is based on thermodynamical functions and was described by Goodrich [43]. Considering the variations in the Gibbs free energy under isothermal-isobaric conditions given by equation (2.5), the excess free energy of mixing ΔG^exc^ can be obtained directly from the *π–A* curves by integration, according to the equation

2.14 $$\Delta G^{\mathrm{exc}}=N_{A}\int_{\pi *}^{\pi }A^{\mathrm{exc}}\;d\pi .$$

A^exc^ is the excess area per molecule ($A^{\mathrm{exc}}=A_{1\ldots N}-A_{1\ldots N}^{\mathrm{id}}$ where $A_{1\ldots N}$ is the mean molecular area in the mixed monolayer and $A_{1\ldots N}^{\mathrm{id}}$ is the area in an ideal mixture described by the additivity rule) at a particular surface pressure, *N_A_* is the Avogadro constant and π* is a surface pressure below which the components can be assumed to be ideally mixed [41,42]. For the two-component system this equation can be rewritten as

2.15 $$\Delta G^{\mathrm{exc}}=N_{A}\left(\int_{\pi *}^{\pi }A_{12}d\pi -X_{1}\int_{\pi *}^{\pi }A_{1}d\pi -X_{2}\int_{\pi *}^{\pi }A_{2}d\pi \right).$$

The value of Δ*G*^exc^ provides information on whether the particular interaction is energetically favourable (Δ*G*^exc^ < 0) or not (Δ*G*^exc^ > 0), while for Δ*G*^exc^ = 0 ideal mixing (or immiscibility) occurs [41,44]. The value of Δ*G*^exc^ allows to conclude about the strength of mutual interactions and possible affinity of the components to each other. In addition, to determine the stability of the mixed monolayers, the total Gibbs energy of mixing can be calculated as

2.16 $$\Delta G_{\mathrm{tot}}^{\mathrm{mix}}=\Delta G^{\mathrm{exc}}+\;\Delta G^{\mathrm{id}},$$

wherein $\Delta G^{\mathrm{id}}=RT\sum_{i=1}^{N}X_{i}lnX_{i}$ and *R* is the gas constant [41].

## 3. The importance of phase transitions in monolayers

Because the phase state and phase transitions of biologically important compounds affect the physical properties of membranes, they seem to be extremely important for their proper functioning. Each phase transition can be easily triggered by relatively small changes in various thermodynamic variables, regardless of whether they occur between two-dimensional phases (in the single-layer state) or are associated with a two-dimensional–three-dimensional transition.

### 3.1. Monolayer state

To obtain insight into the thermodynamics of phase transitions occurring in monolayers, the experimental *π–A* dependencies are measured at different temperatures. This allows to establish the so-called critical temperature, which seems to be crucial in the analysis of bioactive molecules. Moreover, the entropy and enthalpy of the phase transition (Δ*S_t_* and Δ*H_t_*, respectively) can be calculated on the basis of equations (2.10) and (2.11). As discussed in detail in [45], the thermodynamic analysis may be difficult due to the non-ideality of the experimental *π–A* curves (i.e. the plateau in the course of the *π*–*A* isotherm is usually not perfectly horizontal due to the polydispersity of the analysed sample or non-equilibrium compression conditions). This may cause problems with unequivocal establishing of the values of the transition surface pressure and the values of the molecular area corresponding to the beginning and the end of the plateau. However, a methodology suitable even for isotherms without an apparent plateau has been developed and described in [46–48].

One of the most frequent transitions in two-dimensional systems is the conversion of a LE to a LC phase observed for many phospholipids, including DPPC [49], lyso-PC [50], DMPC [49], POPE [46], DMPA [49], DPPG [51,52] and others [53]. This transition has some characteristic features, such as: (i) it can be easily identified microscopically, since the formation of LC phase is manifested by the growth of the characteristic snowflake-like textures observed in BAM images, and (ii) the surface pressure of the plateau region increases with temperature (the slope of *π_t_*–*T* dependence is positive, indicating the first-order transition). Therefore, the calculated Δ*S_t_* and Δ*H_t_* are negative, which suggests that the LE–LC transition is exothermic and that the formed LC phase is more ordered compared with the LE state. The latter has been confirmed by numerous theoretical and experimental studies evidencing conformation with fewer Gauche defects in acyl chains of phospholipids in the condensed state [54,55]. A similar origin (changes in orientation) has also been proposed for the solid–solid transition observed for cholesterol [56].

Another interesting group of compounds are bipolar molecules possessing two (identical or different) polar moieties in their structure. In their case, the plateau in the *π*–*A* isotherms is usually associated with a change in molecular orientation during compression as a result of the detachment of one of the polar groups from the surface of water. As it was reported for isomers of bipolar carboxylic acids, the distance between the polar groups in the molecule is crucial for the temperature behaviour and interpretation of this transition [57]. For example, when an additional polar group is attached to a carbon atom adjacent to another polar group, 2-hydroxycarboxyclic acids act as monopolar entities. When an additional polar group is introduced further in the hydrocarbon chain, the resulting compounds (i.e. 9-, 11- and 12-hydroxystearic acid or 9-hydroxypalmitic acid) show a bipolar character. Namely, in their isotherms there is a wide and flat plateau region, during which the anchoring of the molecule changes (one polar group detaches from the water surface). The effect of temperature on the surface pressure of this plateau (π_t_) is much smaller compared with that estimated for the LE–LC transition. Negative values of Δ*S_t_* and Δ*H_t_* are then obtained; however, their magnitude is greater for amphiphiles with an additional polar group attached at C(2). This suggests that the introduction of a polar group at C(2) modifies the surface occupied by the polar headgroup and leads to a conformational disordering of hydrocarbon chains, which is strongly dependent on temperature. In turn, lower absolute values of Δ*S*_t_ for isomers with polar group introduced at C(9) suggest that monolayers are characterized by well-ordered structure even before the change of surface anchoring manifests itself as a plateau in the *π*–*A* isotherm.

Research conducted on biologically important oxidized cholesterol derivatives (oxysterols), namely 7-hydroxycholesterol epimers (7*α*-OH and 7*β*-OH) showed another interesting issue—the configuration also influences the phase behaviour of such bipolar amphiphiles [58]. Namely, contrary to 7*β*-OH, which isotherm lacks any transition, in the isotherm of 7*α*-OH a clear plateau is visible. The π_t_ decreased with temperature, causing the calculated Δ*S_t_* and Δ*H_t_* values to be positive. Differences in surface behaviour of both epimers have been interpreted as being due to orientation of −OH groups in their molecules; i.e. for 7*β*-OH both hydroxyl groups (at C3 and C7) are situated on the same side of the sterane rings A and B, while for 7*α*-OH on different sides, which induces the lifting off of one hydroxyl group from the water surface upon compression.

When an additional polar group is placed at the opposite end of a rigid, conformationally stable molecule, i.e. at the alkyl chain of a cholesterol molecule, it becomes difficult to anchor both polar groups to the surface. Consequently, the biamphiphile adopts a vertical orientation with one polar group anchored in the subphase and the other facing the air. Surprisingly, in the *π*–*A* isotherms of such compounds, for example 25- and 27-hydroxycholesterol (25-OH and 27-OH), a plateau region is also visible. It has been evidenced by a variety of experimental methods that such compounds undergo—upon compression—a phase transition from a monolayer to reproducible and well-ordered bilayer structures [59,60]. The thermodynamic analysis of *π*–*A* curves pointed to significant differences in the character of the transition for each isomer. Positive values Δ*S_t_* and Δ*H_t_* suggest that the formation of a bilayer from 25-OH molecules is endothermic and irreversible. On the other hand, for 27-OH the process is reversible, and Δ*S_t_* and Δ*H_t_* values are close to zero.

### 3.2. Collapse

The reversibility of monolayer collapse is of great importance in biological processes occurring in lungs, ears and eyes. The lung surfactant (LS) is a mixture of lipids (mainly DPPC and other phospholipids such as unsaturated PCs and PGs) and specific cationic proteins [61]. Functioning of lungs is based on the following processes involving LS monolayers: (i) compression with low surface tension (exhalation), and (ii) film respreading during expansion (inspiration). The latter is possible due to the fact that collapse of LS mixture occurs via a plateau and is reversible. Although pulmonary lipids themselves (DPPC and unsaturated and anionic lipids) play an important role in lowering the work of breathing (details and references can be found in the excellent review by Lee [39]), they cannot provide the required reversibility of the collapse, so that the collapsed monolayer can be reabsorbed during inhalation. This property is achieved in the presence of cationic lung surfactant protein, which changes the mechanism of collapse to reversible folding. Similar phenomenon of collapse reversibility is necessary to occur in normal ears functioning. Surfactants of the middle ear and Eustachian tube are of similar composition to LS, i.e. the main lipid component is DPPC and it also contains specific proteins [62]. This mixture of phospholipids and proteins directly adsorbed to solid mucosal surfaces acts as a relieving agent to reduce the pressure associated with opening a blocked Eustachian tube. Another example is the tear film lipid layer, which is a complex system of lipids (containing 8–16% of lung lipids) and proteins [63]. The reversibility of the repeated full compression and expansion cycles is of utmost importance in preventing film rupture during a blink [64].

## 4. Interactions in multi-component biomimetic systems

In biological systems, mutual miscibility or phase separation plays a key role [65]. On one hand, the phase separation can lead to defects in transport (exocytosis and endocytosis) and fusion processes. In turn, strong attractive forces, which usually result from hydrophobic interactions between apolar parts of molecules, are responsible for the formation of the so-called *surface complexes* [66–68]. These assemblies show significant deviations from ideal behaviour, quantified by highly negative *A*^exc^ and Δ*G*^exc^ values (equation (2.15)). The hypothesis on the formation of surface complexes was used to explain the membrane lateral heterogeneity and determine the composition of the so-called lipid rafts. Additionally, the analysis of interactions was used to investigate the membrane effects of bioactive substances and their activity under physiological conditions. The following section presents the most important examples.

### 4.1. Biomembrane heterogeneity—the concept of lipid domains

A framework of biomembrane bilayer structure contains plethora of lipids with random distribution, acting as a lipid ‘solvent’ for proteins (fluid mosaic model [69]). However, later studies showed that the structure of the membranes is not completely homogeneous. It is currently assumed that biomembranes are heterogeneous structures composed of two phases: an ordered liquid phase, *l*_o_ (which consists of clusters (with a size below 4 nm) or domains (with a size ranging from nano to micrometres) [70]) and a disordered liquid phase, *l*_d_ (formed mainly by glycerophospholipids [71]). The liquid ordered phase can be defined as a gel-like phase of a lipid biomembrane enriched in cholesterol. This phase is characterized by relatively close molecular packing (acyl hydrocarbon chains of lipids are in the all-trans state). In contrast, liquid-disordered phase is a highly fluid state of biomembrane, poor in cholesterol, in which lipids can move freely across the plane of surface. *L*_d_ is usually characterized by irregular packing of lipids. Both states can be distinguished due to the application of biophysical techniques (e.g. nuclear magnetic resonance (NMR) or X-ray scattering) enabling the assessment of the movement and structure of lipids in mixtures [72,73].

**Figure 2.**
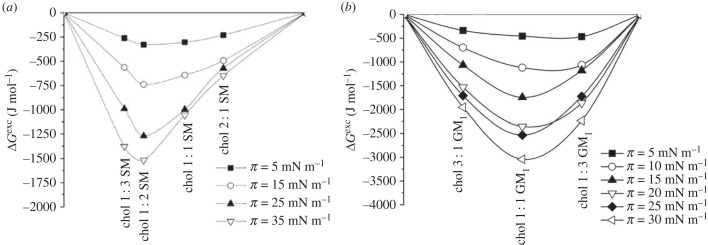
Δ*G*^exc^ plots for: (*a*) cholesterol/sphingomyelin and (*b*) cholesterol/ganglioside mixtures (modified from [78,79] with permission from Elsevier and the Royal Society Publishing.

The term ‘lipid domains’ is closely associated with lipid rafts, which are domains enriched in sterols and sphingolipids. The other ordered assemblies found in biomembranes are formed by ceramides, glycosphingolipids and phosphoinositides (for details, see review article [70]). The formation of lipid rafts is related to interactions between sterols and phospho- and glycolipids. Initially, these domains were identified only with the outer leaflet of eukaryotic cells (plasma membrane). Today it is known that lipid rafts may be also formed in the membranes of bacteria [74] and in the inner membranes, as well as in cytosolic layer of the membrane [75], and are characterized by different composition. However, it is important to emphasize that the topic of lipid rafts is complex and still requires a lot of research. It has been also found that the study of lipid rafts in living cells is difficult, and until now their existence in biological systems has been a matter of controversy. However, as stated in [76], their visualization in living systems is just a matter of time due to the increasing development of imaging techniques. Therefore, lipid rafts are currently studied using models such as Langmuir monolayers. Importantly, the thermodynamic analysis of interactions made it possible to determine the stoichiometry of the raft components. The first suggestions for the possible composition of lipid rafts came from cellular studies of cholesterol homeostasis, which were found to be dramatically influenced by sphingomyelin [77]. This suggested that these two lipids had a strong affinity for each other. Indeed, further studies on interactions in two-component systems confirmed strong affinity of cholesterol to sphingolipids (mainly sphingomyelin and gangliosides [78,79]), which may be explained on the basis of the molecular geometry [80,81]. Specifically, the inverted cone shape of cholesterol (promoting negative curvature structures) fits perfectly with the truncated cone-like shape of sphingomyelin or cone-shape of ganglioside GM_1_ (both forming positive curvature structures) [81]. The interactions analysis in the mixed Langmuir films pointed that the SM/Chol 2 : 1 and GM_1_/Chol 1 : 1 systems show the minimum values of Δ*G*^exc^ (figure 2). Therefore, these mixtures can be considered as artificial lipid rafts, among which the SM/Chol system is used as the simplest model [78,82–85].

In search for other raft-mimicking systems, phosphatidylcholines were added to the SM/Chol model mentioned above, ensuring the fluidity of the matrix accommodating the rafts and meeting the *l*_o_/*l*_d_ phase separation conditions [86–88].

**Figure 3.**
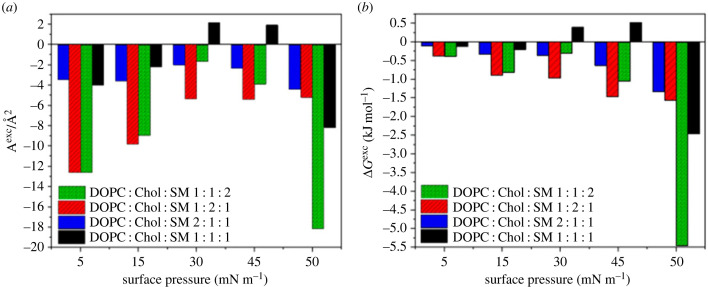
Comparison of (*a*) *A*^exc^ and (*b*) Δ*G*^exc^ calculated at selected surface pressure values for DOPC/Chol/SM systems mixed in different proportions. Adapted under Creative Commons Attribution 3.0 License from [87].

In [87] the mutual proportions in three-component systems were selected taking into account not only the minimum of Δ*G*^exc^ values, but also the ratio of *l*_o_ to *l*_d_ phase present in the biomembrane. In this context, the SM/Chol/DOPC (1 : 1 : 1) system has been proposed as the best lipid raft model. What is interesting, the system with SM in excess seems to be more stable (the lowest value of Δ*G*^exc^, figure 3*b*); however, according to the authors, the amount of fluid matrix in this mixture was underestimated compared with real systems.

Other PC lipids were also included in the lipid raft model: (i) POPC was considered as more biologically relevant [88], while (ii) DPPC—having both saturated chains—was found to be able to form more packed structure with cholesterol [86]. In the literature, four–component models (i.e. PC/SM/Chol/GM_1_ [89] or PG/SM/Chol/GM_1_ [90]) can also be found, but their stability is low. Interestingly, after elimination of GM_1_, the stability of mixed systems increases [89]. It should be noted that the more components a model contains, the more difficult it is to interpret the results. Therefore, the choice of an appropriate system imitating lipid rafts should always be dictated by the best possible optimization of parameters.

### 4.2. Membrane effects of bioactive substances

A bioactive substance present in the body, either through exogenous delivery or endogenous processes, can incorporate into the cellular membrane, changing its physico-chemical properties. This effect can be studied by monitoring the membrane rheological properties (by looking at the membrane compressibility) and/or studying biomolecule–membrane interactions based on the thermodynamic approach.

#### 4.2.1. Changes in membrane fluidity

There are plethora of examples in literature showing the influence of bioactive substances on membrane rheological properties. In this aspect, the Langmuir monolayer technique is one of the best methods to choose.

It is worth noting that the proper functioning of biomembranes depends on their fluidity, which may be influenced by changes in their lipid composition, resulting, for example, from dieting or taking medications or dietary supplements. It is also characteristic of the development of many diseases. (e.g cancer [91] or anaemia [92]). There are numerous examples in the literature showing disturbances of both stiffening and fluidization of membranes by drugs (for a review see [25] and references therein), which can be further considered to elucidate their biological activity. An interesting example can be provided by local anaesthetics, which were found to insert into simplified models of selected cell membranes, causing their fluidization [93]. Such an effect on nerve cells lipid membrane can change conformation of channel proteins responsible for transport of sodium ions, explaining in this way their anaesthetic activity. However, the action of anaesthetics is not limited solely to nerve cells. These drugs also affect other types of cells and organelles (e.g. erythrocytes, and mitochondria), causing severe side effects, which can be related to their effect on membrane rheological properties.

It is important to mention that the fluidity of cell membranes in pathological processes changes versus physiological conditions, as indicated above for the case of cancer or some infections (e.g. in malaria [94]). Generally, in most pathological processes, the fluidity of cell membranes increases, which may explain the selective effect of some drugs (for example cyclosporin A or synthetic anti-tumour lipids) on pathologically changed cells, which, being more fluid, more easily incorporate drug molecules, while healthy cells—with a more rigid membrane—are resistant to their incorporation.

#### 4.2.2. Thermodynamic analysis of biomolecule–membrane interactions

Analysis of interactions between biomolecules and membrane components with excess thermodynamic functions allows for: (i) verification of hypotheses regarding the mechanism of action of bioactive substances, (ii) identification of their molecular targets, and (iii) identification of toxic effects and methods of their elimination. This part presents selected examples of research problems solved by thermodynamic analysis of interactions.

##### 4.2.2.1. Membrane activity of amphotericin B

**Figure 4.**
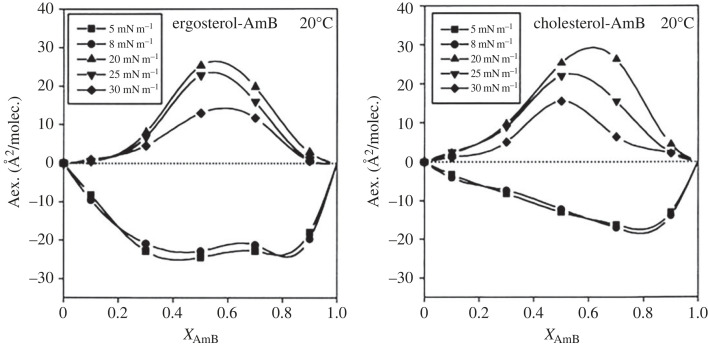
Excess area of mixing *A*^exc^ for ergosterol- or cholesterol-containing monolayers in function of AmB content. Adapted from [66] with permission from Elsevier.

Amphotericin B (AmB) is a polyene macrolide antibiotic with broad anti-fungal activity. Its mode of action is related to the ability to create pores (transmembrane channels) that disrupt the structure and function of biomembranes, leading to the leakage of ions and other important elements from the cell. Based on biological studies, it was postulated that sterols are involved in the construction of pores by forming complexes with AmB [95]. To verify this hypothesis, interactions were examined in Langmuir monolayers from AmB mixed with a sterol characteristic of mammals (cholesterol) and fungi (ergosterol). Negative deviations from ideality were observed at low surface pressures, confirming the formation of stable hydrogen-bonded surface complexes with vertically oriented sterols and horizontally oriented AmB molecules [66]. This indicated that sterol molecules act as ‘glue’ binding AmB macromolecules, which may lead to the formation of pores. The stronger interactions of AmB with ergosterol than with cholesterol (figure 4), expressed in *A*^exc^−*X* plots, explained its lower toxicity toward mammalian host cells compared with fungi [66].

Further insight into AmB selectivity was achieved by taking into account results for DPPC-containing system [96]. Thus, in the case of the cell membrane, the three kinds of interactions should be considered: sterol/phospholipid, AmB/sterol and AmB/phospholipid. For the fungal membrane, the interactions between ergosterol and phospholipid are very weak [97], therefore ergosterol and phospholipid compete with each other to form complexes with AmB. Since the affinity of AmB to ergosterol is higher than to the phospholipid, pore formation is induced in the fungal membrane. In the case of the mammalian membrane, the interactions between cholesterol and the phospholipid are very strong [67] and, consequently, the amount of free cholesterol (and also phospholipid) is low [98]. As a result, the degree of cell damage is much less than in the fungal membrane. The review article by Kamiński [99] summarizes the results obtained so far on the mode of action of AmB, using traditional techniques, taking into account various aspects that may be also important (e.g. aggregation of AmB). Readers interested in recent updates on modern research concerning mode of action of AmB and other polyene antibiotics are recommended to see recent review article [100].

##### 4.2.2.2. Membrane targets of anti-tumour drugs

**Figure 5.**
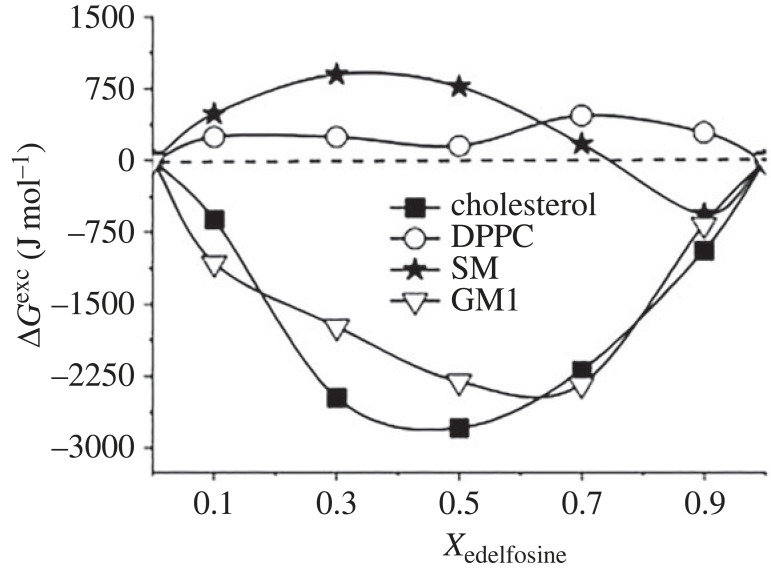
Δ*G*^exc^-X_edelfosine_ plots for mixtures of edelfosine with chosen lipids at 30 mN m^−1^. Modified from [104] with permission from ICE Publishing.

Thermodynamic analysis of the interactions enabled to establish membrane targets of synthetic anti-tumour lipids (ATLs), which structurally resemble natural phospholipids [101,102]. Among ATLs, the most known are edelfosine (1-O-octadecyl-2-O-methyl-*rac*-glycero-3-phosphocholine) and alkylphosphocholines (APCs), e.g. hexadecylphosphocholine (miltefosine). Unlike standard anti-cancer drugs, their target is the cell membrane, not DNA (such as for cisplatin). Therefore, it was crucial to clarify which membrane component(s) can play a key role in cell targeting (for a review, see [103]). Figure 5 shows the comparison of Δ*G*^exc^ values for two-component systems of edelfosine and main membrane lipids.

The strong attractive interactions with cholesterol and GM1 indicate that these molecules are important in the mode of action of edelfosine, while neither phospholipids nor sphingomyelin appear to be important in this respect due to weak interactions with a tendency to phase separation. The observation of the formation of edelfosine/Chol 1 : 1 surface complexes [105] was important for understanding the results obtained in cell cultures, showing that the presence of excess cholesterol (in the incubation medium or in the membranes) reduces drug absorption [106,107]. This effect can be explained by a reduction in the amount of ‘free’ edelfosine by its immobilization in complexes with cholesterol. In consequence, only a part of the unbound drug can freely penetrate the membrane, exerting its biological activity. This finding has important consequences. Firstly, the pharmacological activity of edelfosine can vary among different types of cells, depending on cholesterol level in the plasma membrane [106–109]. Secondly, the formation of cholesterol–drug complexes accounts for the observed lower haemolysis of erythrocytes in cholesterol-containing edelfosine formulations [110] as the amount of free drug responsible for haemolysis is reduced by its binding to cholesterol. On the other hand, the analysis of interactions between edelfosine and GM_1_ was helpful in understanding drug efficiency in brain tumours. As gangliosides are overexpressed in outer membrane leaflet during tumour progression, they are perceived as membrane targets for therapeutics. In order to get insight into the edelfosine activity, a glioblastoma model membrane (SM/Chol mixed in 0.2 molar proportion) containing different GM_1_ content were prepared [111]. Interestingly, the drug was effective only at a high concentration of ganglioside (above 10% in the model membrane). This explains why edelfosine exerts its pharmacological action in the advanced stage of cancer. Interesting results were also obtained for alkylphosphocholines. Two of them (hexadecylphosphocholine (miltefosine), HePC; and octadecylphosphocholine, OcPC), similarly to edelfosine, formed surface complexes with cholesterol of strictly defined stoichiometry (1 : 1), while erucylphosphocholine, ErPC was strongly bound to cholesterol practically in the whole range of mole fractions [112]. In this case there is no excess of ‘free’ drug that could cause haemolysis because almost all ErPC molecules form stable complexes with cholesterol. Indeed, biological studies have shown that ErPC has the lowest haemolytic properties among all other APCs and it can be administered both by injection and orally, which significantly improves its therapeutic effect [101].

**Figure 6.**
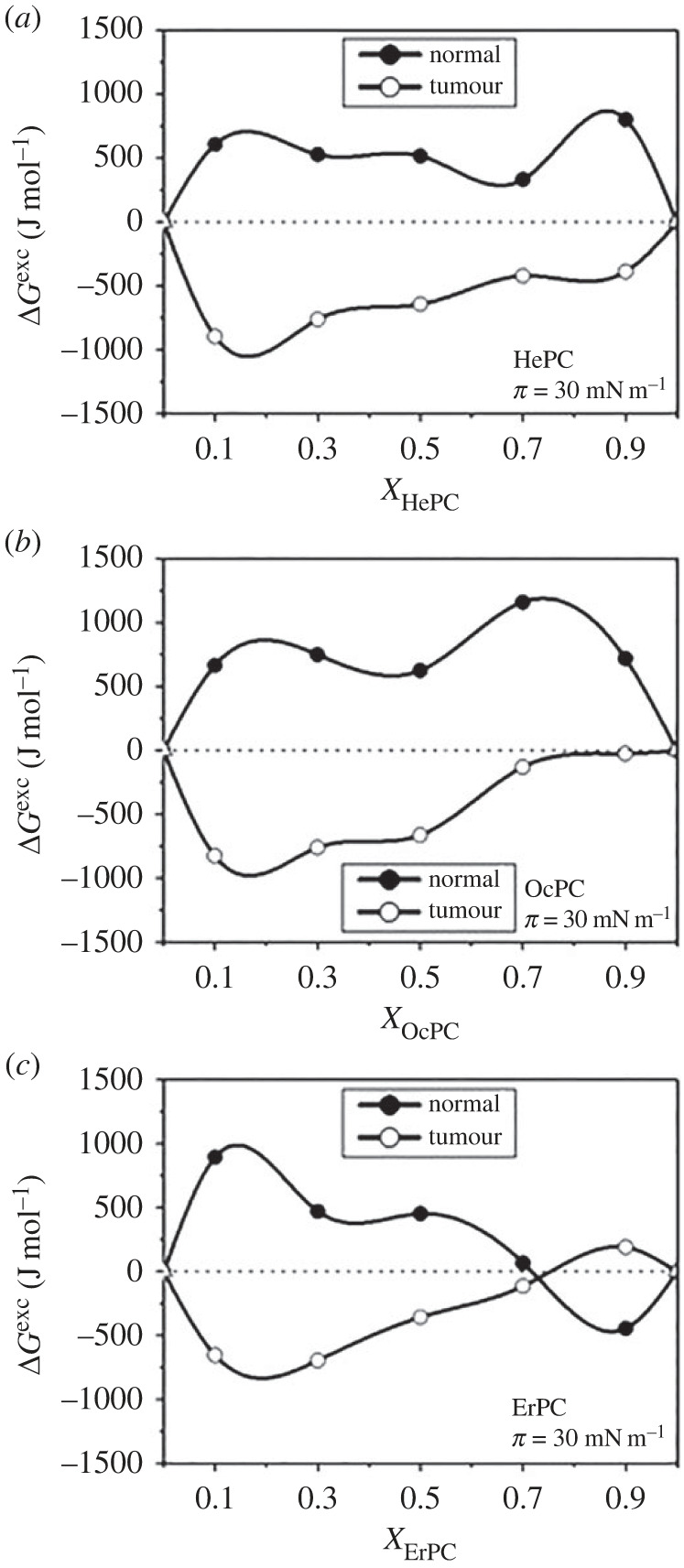
Δ*G*^exc^–XAPCs plots for systems mimicking normal and tumour membrane treated with APCs at 30 mN m^−1^. Adapted under Creative Commons Attribution License from [112].

Thermodynamic analysis was also important in understanding the selective effect of APCs on tumour cells. The drugs (HePC, OcPC, and ErPC) were incorporated into two types of monolayers: the white blood cell membrane model (Chol/DPPC 2 : 3), and leukemic cell membrane model (Chol/POPC 1 : 4). It has been shown that APCs affect both types of model membranes, but in different ways (figure 6). Based on Δ*G*^exc^ values, it was found that the insertion of APCs molecules into the cholesterol/POPC monolayer—from a thermodynamic point of view—is much easier than the incorporation into the cholesterol/DPPC film. It is also related to (i) different molecular organization of the monolayer mimicking tumour and normal cell membrane (the Chol/DPPC film is more ordered and condensed than the Chol/POPC system), and (ii) a stronger affinity of cholesterol to DPPC compared with POPC [113,114]. Therefore, it can be concluded that the normal cell membrane is a natural barrier preventing APCs molecules from penetrating into healthy cells and explains the high selectivity of APCs (confirmed by biological experiments) [112]. Other studies have also shown a strong affinity of APCs (especially ErPC) to the prostate cancer membrane (the therapeutic effect has been associated, among others, with the fluidization of the tumour membrane) [115].

Another, group of anti-cancer compounds, in which activity was found to occur at the membrane level, are pentacyclic terpenes. For example, betulinic acid (BA) has been shown to be highly selective in cancer cell lines as well as on animal models [116,117], but it was not effective against bacteria. Studies involving Langmuir monolayers have helped provide insight into this issue by revealing that the primary membrane target of pentacyclic terpenes is cardiolipin (CL) [118,119]. However, different cells contain CLs with different chain lengths and unsaturation, and their interactions with a particular pentacyclic terpene may differ, as reflected in the differential selectivity of these compounds. Specifically, for BA it was shown that interactions with bacterial CL (14 : 0) were of ideal character, while system mixed with bovine heart CL (containing mainly 18 : 2) was immiscible in a wide range of molar concentrations. By contrast, the strong affinity to CL (18 : 1) was found. As this lipid is characteristic for lymphoblasts, it can be considered as a molecular target for BA [118].

##### 4.2.2.3. Interactions of oxysterols in membrane depend on their molecular orientation

Oxysterols are biologically crucial cholesterol derivatives possessing additional polar group in their structure. The individual representatives of this group show unique phase behaviour in single-component monolayers (for details see §3). The latter influences also their behaviour in multi-component systems. For example, in binary mixtures containing 7-hydroxycholesterol epimers and SM (7*α-*OH/SM and 7*β-*OH/SM), the stereostructure-dependent difference in the strength of mutual interactions was shown [120]. Although negative deviations from ideality were observed in both mixed systems, in the case of 7*β-*OH the interactions were much weaker (Δ*G*^exc^ ≈ −500 J mol^−1^) and constant within all studied molar fractions and surface pressures (except for π ≤ 5 mN m^−1^). In contrast, for 7*α-*OH the interactions were much stronger and dose dependent (with minimum at −1250 J mol^−1^ for an equimolar mixture). The reported differences were explained by the formation of hydrogen bonding between hydroxyl groups of SM and 7*α-*OH, whereas in the case of 7*β-*OH this process was hindered from geometric reasons [120].

Interesting surface behaviour was also observed for another oxysterol (25-OH) in mixtures with sphingolipids (GM_1_ and SM). The analysis of Δ*G*^exc^ dependencies showed that 25-OH is arranged in surface complexes with both sphingolipids. Additional information from the *π*–*A* isotherms complemented with BAM, PM-IRRAS and MD simulations revealed that in the 25-OH/GM_1_ system, two types of surface complexes are formed since 25-OH is anchored in water with C(3)-OH or C(25)-OH. On the other hand, SM imposes one specific orientation of 25-OH: by anchoring with C(3)-OH in water [79]. The interactions between sphingolipids and cholesterol or 25-OH may have important biological implications. Namely, the strength of interactions in Chol/GM_1_ versus 25-OH/GM_1_ mixtures is similar, therefore in natural systems these sterols can compete for the interaction with GM_1_. On the other hand, weaker interactions occur in Chol/SM versus 25-OH/SM system. Therefore, 25-OH may easily replace Chol in model lipid raft (SM/Chol 2 : 1), changing its surface and rheological properties, which may result in the dysfunction of these domains [120].

For systems composed of 25-OH mixed with membrane phospholipids characteristic for the outer (PC) and cytosolic (PE) leaflet it was shown that the interaction strength is primarily determined by the type of phospholipid polar head [121]. Namely, strong, attractive interactions leading to the formation of surface complexes were observed for 25-OH/PC, while weak or repulsive interactions occurred in 25-OH/PE systems. The saturation of the phospholipid hydrocarbon chains is crucial for the structure of the surface complexes formed. In the case of 25-OH/DPPC systems, two types of surface complexes were postulated (25-OH is anchored with C(3)-OH or C(25)-OH in water). In contrast, for unsaturated PC (25-OH/DOPC), only one specific orientation of oxysterol was shown (anchoring with C(3)-OH in water). All these indicate that the transport of 25-OH between the membrane leaflets can proceed without changing the orientation of oxysterol molecule, which is thermodynamically favourable [121]. This explains significant differences in the transmembrane transport rate of 25-OH and other chain-oxidized oxysterols compared with their ring-oxidized analogues or cholesterol [122,123].

##### 4.2.2.4. Fatty acids and phytosterols in the regulation of plasma cholesterol level

To better understand the physiological effects of unsaturated (UFA) and saturated (SFA) fatty acids on plasma cholesterol levels, a thermodynamic miscibility analysis approach was used. Results obtained for two-component Chol/UFA and Chol/SFA systems revealed that the hypocholesterolemic effect depends on the miscibility and strength of interactions with cholesterol. Namely, Chol/SFAs mixtures showed immiscibility [124] (based on π_c_ analysis; see §2.2) while systems of Chol/UFAs were found to be miscible, and strong interactions, suggesting formation of surface complexes, were confirmed by low Δ*G*^exc^ values [125]. The hypocholesterolemic effect was linked to immobilization of cholesterol in strong attractive interactions with UFAs. In this way, the level of ‘free’ plasma cholesterol is reduced. To confirm this, a reference drug, known for lowering cholesterol absorption and its plasma level (stigmastanil phosphorylcholine (SPC)), was studied in mixtures with cholesterol [126], and the results were compared with the system with polyunsaturated fatty acid (arachidonic acid, ARA). In both investigated systems (Chol/SPC and Chol/ARA) the interactions were found to be strong and attractive, confirming the relationship between the strength of UFA–cholesterol interactions and their hypocholesterolemic activity. Further experiments involving a wider variety of UFAs suggest that their potency to exert hypocholesterolemic effects (quantified with Δ*G*^exc^ values) depends on their structure (the number and position of double bonds in the hydrocarbon chain) (for review, see [127]).

The effect of UFAs and other hypocholesterolemic substances, i.e. phytocompounds (plant stanols and plant sterols) was also studied on the membrane level. In hypercholesterolemia, excess cholesterol accumulates in cell membranes, increasing membrane stiffness, and leading to pathological processes. It was shown that this process can be reversed by introducing UFAs from the diet. In this way, UFAs protect the membrane against changes in its biophysical properties and normalize its fluidity and permeability, which is required for normal functioning [127]. In this context, thermodynamical analysis for model membranes (Chol/POPC and Chol/POPC/SM) treated with phytocompounds showed weakening of interactions between membrane components [128,129]. Moreover, the presence of phytochemicals in the membrane was found to compensate strong condensing effect caused by cholesterol, similarly to UFAs.

## 5. Conclusion

In this review, we demonstrate that thermodynamic analysis of Langmuir monolayers is a powerful tool for describing the behaviour of physiologically active compounds in a membrane environment. Many examples of extensive analysis of thermodynamic parameters, such as mixing and excess functions, have enabled precise characterization of biomolecule–membrane interactions and allowed insight into the action of many biomolecules without the need to perform time-consuming measurements with complementary surface-sensitive analytical techniques. This knowledge can lead to easier analysis of the behaviour of biomolecules and thus explanation of their mode of action using a simple laboratory tool.

## Data Availability

This work did not require ethical approval from a human subject or animal welfare committee.

## Data accessibility

This article does not contain any additional data.
